# Analysis of the policymaking process in Burkina Faso’s health sector: case studies of the creation of two health system support units

**DOI:** 10.1186/s12961-017-0173-0

**Published:** 2017-02-13

**Authors:** Andre Zida, John N. Lavis, Nelson K. Sewankambo, Bocar Kouyate, Kaelan Moat, Jessica Shearer

**Affiliations:** 1 Clinical Epidemiology and Biostatistics Unit, Makerere University College of Health Sciences, PO Box 7072, Kampala, Uganda; 2 Ministry of Health, 01 PO Box 7009, Ouagadougou 01, Burkina Faso; 3 McMaster Health Forum, Centre for Health Economics and Policy Analysis, Department of Clinical Epidemiology and Biostatistics, McMaster University, 1280 Main St. West, MML-417, Hamilton, ON L8S 4L6 Canada; 4 Department of Political Science, McMaster University, 1280 Main St. West, MML-417, Hamilton, ON L8S 4L6 Canada; 5 McMaster Health Forum, Centre for Health Economics and Policy Analysis, and Department of Clinical Epidemiology and Biostatistics, 1280 Main St. West, MML-417, Hamilton, ON L8S 4L6 Canada; 6 PATH, PO Box 900922, Seattle, WA 98109 United States of America

**Keywords:** Health policy, Policymaking, Agenda setting, Health system financing, Burkina Faso

## Abstract

**Background:**

Burkina Faso has made a number of health system policy decisions to improve performance on health indicators and strengthen responsiveness to health-related challenges. These included the creation of a General Directorate of Health Information and Statistics (DGISS) and a technical unit to coordinate performance-based financing (CT-FBR). We analysed the policymaking processes associated with the establishment of these units, and documented the factors that influenced this process.

**Method:**

We used a multiple-case study design based on Kingdon’s agenda-setting model to investigate the DGISS and CT-FBR policymaking processes. Data were collected from interviews with key informants (n = 28), published literature, policy documents (including two strategic and 230 action plans), and 55 legal/regulatory texts. Interviews were analysed using thematic qualitative analysis. Data from the documentary analysis were triangulated with the qualitative interview data.

**Results:**

Key factors influencing the policymaking processes associated with the two units involved the ‘problem’ (problem identification), ‘policy’ (formation of policy proposals), and ‘politics’ (political climate/change) streams, which came together in a way that resulted in proposals being placed on the decision agenda. A number of problems with Burkina Faso’s health information and financing systems were identified. Policy proposals for the DGISS and CT-FBR units were developed in response to these problems, emerging from several sources including development partners. Changes in political and public service administrations (specifically the 2008 appointment of a new Minister of Health and the establishment of a new budget allocation system), with corresponding changes in the actors and interests involved, appeared key in elevating the proposals to the decision agenda.

**Conclusions:**

Efforts to improve performance on health indicators and strengthen responsiveness to health-related challenges need focus on the need for a compelling problem, a viable policy, and conducive politics in order to make it to the decision agenda.

## Background

Public policy is defined as a course of action or inaction chosen by public authorities to address a given problem or interrelated set of problems [1]. Over the last two decades, Burkina Faso’s government has made many decisions about health system policy. These public policy decisions, which have included establishing new policy and performance management units, were typically intended to improve population health and enhance the responsiveness of the health system to important health-related challenges [2] such as high maternal [3] and neonatal [4] mortality. Decision-making is a complex subject, however (for example, [5–7]), and few of these decisions have been evaluated in terms of how and why the decision was made [8]. Understanding the process of policy reform is essential to identify what is needed to move health system issues onto the decision agenda and then implement change.

Many of Burkina Faso’s most significant health reforms occurred under the 2001–2010 National Health Strategic Plan. In 2005, the midterm evaluation of the implementation of the plan found the country was not on track to meet Millennium Development Goals (MDGs) by 2015. This resulted in greater emphasis being placed on system performance, and led to important policy changes. These changes included establishing performance contracts with health districts under the Health Development Support Program, launching a new process for contracting with non-governmental organisations (NGOs) and other private sector actors, and extending subsidies for births and emergency obstetric and neonatal care [9]. Despite these actions, many concerns were raised; for example, the operationalisation of the National Health Information System remained inadequate [10]. This system, characterised by poor sub-system coordination, low quality of available data, and insufficient human and material resources, made timely access to information difficult, and therefore affected the ability to monitor progress towards the MDGs.

These concerns, along with other challenges associated with the need to monitor and evaluate the 2011–2020 National Health Strategic Plan (accelerated growth and a sustainable development strategy) and the MDGs, highlighted a need for the Ministry of Health to initiate measures to strengthen health information and performance systems. The creation of a General Directorate of Health Information and Statistics (DGISS) was part of this, along with a technical unit to coordinate performance-based financing.

The DGISS, established in 2009, was intended to address the Ministry of Health’s concerns and make recommendations to the National Statistics Council and development partners. A key component of the directorate was meant to be a central unit with sufficient capacity and skills to ensure the successful development of the health information system. This also reflected the need to ensure the coordination and timely production of quality health statistics drawn from various levels of the health system. The objectives of this central unit focused on the development and coordination of the National Health Information System, production and dissemination of health statistics, coordination and promotion of the development of databases and computer applications, and the coordination and promotion of health research that drew on the databases.

**Fig. 1:**
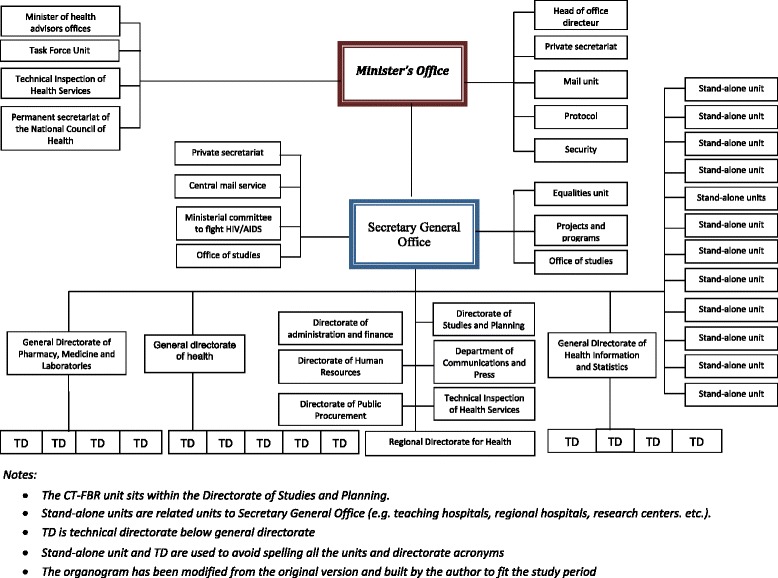
The structure of the Burkina Faso Ministry of Health, showing the location of the two units studied

The second unit, a technical unit to support the coordination of performance-based financing (CT-FBR), was also established in 2009, and was funded to support the quantitative and qualitative improvement of healthcare delivery through a contractual approach. Specifically, this involved the payment of incentives based on provider performance, with funds only disbursable if providers met specific quality standards. Although its broad goals were to contribute to improving the health status of the population and the performance of health services, the CT-FBR was also intended to improve the availability of quality services, increase the use of health services, strengthen the role of operational-level actors in the organisation and delivery of healthcare, and strengthen health system governance. Under the performance-based financing system, district- and hospital-level providers were to receive incentive payments based on a set of quality indicators related to care and prevention. To measure performance, the CT-FBR developed a list of indicators, along with a manual and user guide, and collected data on these indicators to evaluate performance. Figure 1 shows the location of these two units in the overall structure of the Ministry of Health.

The creation of the two units was intended to support initiatives adopted to implement Burkina Faso’s National Health Strategic Plan and achieve the MDGs by 2015 [11]. At least part of the success in attaining the MDGs depended on the ability of these units to perform their assigned duties. Why and how the Ministry of Health decided to place the creation of these two units on the decision agenda, and what factors were found to support implementation, could help other countries grappling with similar challenges.

### Study objective

This study aimed to (1) analyse the policymaking process in Burkina Faso’s health sector related to the establishment of two units; (2) document the factors leading to these units appearing on the decision agenda and supporting the decision to implement; and (3) identify factors and issues that supported the implementation process.

We wanted to understand how the Ministry of Health made the decision to create new organisational units within the health system, and then went about the process of creating them.

## Methods

### Study design

We used a multiple case study design [12]. This design was appropriate as it enabled efficient demonstration of a complex phenomenon. Case studies are useful when it is impossible to dissociate the studied phenomenon from its context [13]. Using more than one case study also permits cross-case analyses that may result in relevant conclusions [14]. We interviewed various players to determine the factors that resulted in the creation of the two units being placed on the Ministry of Health’s decision agenda, as well as the steps involved in the policymaking and implementation process [14]. In this particular case, we recognised that there might be some overlap between the two units, because they were established in the same year, and the establishment processes involved some of the same people. We were therefore careful to look for any overlaps that might affect the establishment of similar units elsewhere.

We used the framework set out by Zmirou-Navier [14] to organise the data and guide the analysis. This framework provides an explanation of the different steps, both concurrent or successive, throughout the process of decision-making on matters related to environmental risk. It also justifies why evaluation, followed by risk assessment management, is required for a clear delineation of the roles of the various actors so that responsibilities can be clearly attributed.

### Conceptual framework

This paper uses Kingdon’s [15] framework to analyse data, focusing primarily on what Kingdon called the agenda-setting stage of the policymaking process. This stage describes the process by which issues rise in prominence in the minds of decision-makers, eventually landing on the formal agenda for government consideration.

Kingdon’s framework [15] was developed to explain how this process happens in reality, and is based on three ‘streams’, namely problems, policies and politics. The problem stream describes events such as a change in an indicator, a focusing event, or feedback from current programs that caught decision-makers’ attention. They are defined as problems requiring government action either because they violate important values or engender unfavourable international comparisons. The policy stream refers to the generation of policy proposals to resolve an identified problem. Policy proposals are necessary for policy decisions, and surface through diffusion of ideas in the policy arena, feedback relating to current policies, or communication and persuasion. Technical feasibility, coherence with national values and mood, and the possibility of anticipating future constraints (budget constraints and public and politician acceptability) affect the survival of policy proposals in a context of multiple serious considerations. Hidden participants (e.g. researchers, civil servants, development partners) are involved in this process. The politics stream is concerned with political events such as swings in the national mood, changes in the balance of organised forces, or events within government. The government agenda or “*list of subjects to which governmental officials and those around them are paying serious attention*” [15] is influenced by visible participants (e.g. the Prime Minister, journalists, interest group leaders) and by events in the problem or politics streams. The joining of these streams influences the decision agenda or “*the lists of subjects within the governmental agenda that are up for an active decision*” [15].

The policymaking process involves many players who, as emphasised by Axelrod [16], act according to their institutional position and resources at their disposal, as well as their own preferences. These actors play important roles in the policymaking process.

### Case study selection

A number of new units have been established in the last 10 years across the Ministry of Health, including eight new health districts. The cases were chosen using criteria that allowed comprehensive understanding of how the policymaking process resulted in the creation of new organisational units within the Ministry of Health [17, 18]. The inclusion criteria used to identify the cases were (1) a focus on units that acted as managing or coordinating entities; (2) a focus on recent policy decisions so the likelihood of robust data sources was high, particularly key informants still in their Ministry of Health roles (a criterion that was included to address concerns about high staff turnover); (3) the existence of legal texts such as decrees, administration letters, or ministerial orders that authorised implementing the policy into practice; and (4) decisions made were actually implemented.

The secondary criterion for case selection was the principal investigator’s ability to access information and key informants related to that case. The two cases selected were promising, given the primary investigator had worked within the DGISS since its inception, and had close professional ties with those working in the CT-FBR. It was therefore likely that policymakers would be prepared to be more open during interviews, although there was also a danger that they might assume the researcher had more knowledge than was the case, and not provide sufficient detail, or ask for information to be kept confidential unnecessarily.

The principal investigator effectively acted as a participant observer during the study [19]. Participant observation is the process enabling researchers to learn about the activities of people under study in their natural setting, through observing and participating in those activities. The principal researcher had 11 years of work experience in the health system and had been involved in several of the policymaking meetings about setting up the new units. He was therefore aware of some of the details of the process, including trips abroad to study other units. He was also able to identify most of the stakeholders involved. Previous studies [20, 21] have noted the advantages of using participant observation as a method of data collection.

It should be noted that this study did not focus on the missions, outputs, outcomes, or impact of the units (where the principal investigator may have encountered conflicts of interest), but rather the decision-making process involved in their creation, moving towards the process of their implementation.

### Participant selection

For each case, potential interviewees were identified and invited to participate in in-depth interviews using a stakeholder purposive sampling approach. This approach is recommended in qualitative studies, as noted by Charmaz [22]. Palys [23] suggested “*… stakeholder sampling is useful in the context of evaluation research and policy analysis, which involves identifying who the major stakeholders are who are involved in designing, giving, receiving, or administering the program or service being evaluated …*” (pp. 697–8). In our study, participants were identified by their position in the health system and how involved they were in the relevant policymaking processes, and particularly the agenda-setting processes [24, 25], by looking at lists of attendees at key meetings about the establishment of each unit. There was also an element of convenience, as interviewees also needed to be still working in the health system.

In total, 28 key informants were interviewed across three categories; 14 participants had a close relationship with agenda-setting related to the National Health Information System (DGISS unit), 8 were associated with agenda-setting related to the CT-FBR, and 6 were from the Ministry of Health’s general administration. A full list of participants, suitably anonymised and categorised by job role, is included in Table 3: Appendix 1.

### Data collection

Data were collected using different methods and from different sources. Data sources included interviews, published literature, trip reports related to the cases, minutes of meetings, policy documents, strategic plans, and relevant operational plans and flowcharts.

In the document analysis, we investigated 2 health sector strategic plans, 230 action plans, 7 trip reports, the Burkina Faso Ministry of Health website, journal articles, 55 regulatory texts, local media articles, and 3 organisational flowcharts.

Individual in-depth interviews were conducted between March and September 2013 by the principal investigator. Interviewees were given a consent form, and the interviews used a guide based on Kingdon’s agenda-setting model [15]. Interviews were conducted in-person in French, and primarily took place in Ouagadougou, Burkina Faso’s capital city. An English translation of the interview guide is included as Appendix 2. One policymaker was interviewed via Skype as he was out of the country during the period of field research. Notes were taken during the interviews, which were audio-recorded with participants’ consent. Interviews lasted 40 minutes on average. Informed consent was obtained from all participants.

### Data analysis

Interview data were transcribed and coded based on a pre-determined coding guide that followed the agenda-setting model [22] and interview guide. This allowed us to categorise the commonalities between the interviews [22]. The interviews were analysed using thematic qualitative analysis [26] and the grounded theory substantive coding process [22]. We read the interview transcripts and field notes several times looking for topics that were frequently mentioned, and summarised these in brief phrases, such as ‘key players in unit creation’, ‘date of unit creation’, ‘sources of funding’ and ‘policymaking steps’. The phrases were listed in the order they appeared in the interviews in a table in Microsoft Word (2010). We then looked for logical groupings and linked these to Kingdon’s agenda-setting framework [15, 22]. Documents and reports relating to the study purpose were also systematically tabulated and analysed against the emerging topics to ensure that the data obtained during the document analysis were triangulated with the qualitative results of interviews [27, 28] until saturation was reached.

Information such as the timeline leading to the creation of the two units was added after the interview and document analysis, and the completed table was sent to the policymakers involved to ensure that correct information had been captured, and additional information was included where possible.

## Results

### Case study 1: DGISS

The process of creating the DGISS was influenced by factors in each of the problems, policies and politics streams.

#### Problems

Defining the problem is one of the first actions in analysing a public policy issue [15]. However, understanding a public policy problem also requires an understanding of the timeline under which the problem emerged and was considered. Table 1 shows the timeline for the events leading up to the establishment of the DGISS.

**Table 1:** Events linked to the creation of the General Directorate of Health Information and Statistics (DGISS)

Year	Event
1989	Center for Epidemiological Surveillance created
1991	Center for Epidemiological Surveillance transformed into the Center of Health Information and Epidemiological Surveillance
2000	General Assembly on Health held
	Health system reforms proposed to improve health indicators and increase the health system’s responsiveness to important health-related challenges
	Healthcare services decentralised
	National strategies and policies (e.g. the MDGs, Strategic Framework for Poverty Reduction, National Health Policy, and the National Health Strategic Plan) adopted by government
2001	Epidemiology training network for students created at the National School of Public Health
	Implementation of the National Health Strategic Plan
	Implementation of the Health Development Support Program
2003	Statistics Master Plan adopted
	Order for the organisation of the Directorate of Studies and Planning for Health (December 2003)
2005	Midterm evaluation of the National Health Strategic Plan
2006	National Program for Strengthening Statistics System Capacity created to produce more reliable and better-quality data with the support from technological and financial partners (e.g. European Commission)
	Report on the quality of routine data drafted
	Evaluation of the National Program for Strengthening Statistics System Capacity
	Report on the quality of routine data drafted
2007	New Prime Minister nominated
	Cabinet shuffle resulting in an economist appointed to lead the Ministry of Health
	New law adopted (Law No. 012-2007/AN of May 31, 2007 on the organisation and regulation of statistical activities) to organise and supervise public sector statistical activities
	Midterm evaluation of the Health Development Support Program
	National Statistics Council created
	Directorates or units in charge of statistics created within Ministries to strengthen statistical production and culture
	Numerous statisticians and demographers recruited and trained to build capacity
	The final year for drafting the health map (3rd edition) by the statistics and the planning department with Help-Mapper software
	Decree No. 2007-390/PRES resulted in announcement of law No. 012-2007/AN of May 31, 2007, on the organisation and regulation of statistical activities (2007 Statistics Law)
2008	Ministry of Health departments reorganised by type
	New flowchart for the Ministry of Health drafted
2009	Decree No. 2009-104/PRES/PM/MS on the organisation of the Ministry of Health (including the creation of the Directorate-General of Health Information and Statistics—Article 33) enacted

*MDGs* Millennium Development Goals

The issue of health information and its coordination and management has a history of cycling on and off governmental agendas, typically driven by particular problems and focusing events [29]. For example, in 1989, the Burkina Faso Center for Epidemiological Surveillance was created to support the tracking of health statistics information, and enable publication of more accurate annual health statistics [30]. In 1991, this centre became the Center for Health Information and Epidemiological Surveillance. This extended the unit’s goal to include tracking of health information. However, feedback from users of health information, statistics and indicators highlighted that data were not available in a timely manner to support decision-making [31]. The waiting time for updated indicators in the annual health statistics was as long as 2 years [31]. In 2000, in response to this feedback, the General Assembly on Health recommended creating a separate unit. It was suggested that the unit would improve the National Health Information System and the availability of indicators to inform evidence-based decisions and support the implementation of international and national policies [31]. However, there was little appetite to create a separate unit at this time, and from 2000 to 2008, the health information system work was done in the Directorate of Studies and Planning at the Ministry of Health.

In 2005, the midterm review of the National Health Strategic Plan highlighted the lack of coordination, human resources and poor data quality related to health information systems and indicators [32]. According to the 2005 Health Metric Network evaluation [33], the lack of published information and poor dissemination of health information and indicators was explained by the lack of National Health Information System management.

The midterm review of the National Health Strategic Plan created pressure to create a technical management structure charged with influencing the National Health Strategic Plan and coordinating National Health Information System sub-systems. Interview participants noted that certain indicators and feedback from current operations revealed several problems that promoted the creation of a directorate to manage the National Health Information System:

“*The annual statistical report, which is the reference document for the Health Information System, was conveniently published in June or July of the following year, instead of being published at most within the first three months of the following year; the private sector indicators were underestimated in the annual statistical report; each health program collects its indicators separately and several regional directors of health complain about the high number of data collection formats and repeated data collection of the same information by several different sources*.” [Project coordinator]

The problem was highlighted again in 2006 through a quality indicator. The Burkina Faso Ministry of Health published a routine data quality assessment showing that 36% of the data collected was not of sufficient quality [31, 34]. In April 2006, the National Program for Strengthening Statistics System Capacity (ARCS) was created with support from several development partners (e.g. the European Commission and WHO) to support the production of more reliable and better-quality statistical data [35]. The ARCS conducted a baseline survey confirming many of the data-related problems previously identified, and identified a number of areas for improvement, including (1) the lack of support staff and the strong mobility of executive personnel (including statisticians, demographers, computer scientists, and public health doctors) made innovative projects difficult to conduct; (2) the absence of regulatory texts that specified the mechanisms for coordinating health information system actors; (3) the lack of a permanent survey system (both in health services and population health) for periodic indicators to monitor the MDGs, National Health Strategic Plan, and other health policies and programs, leading to redundant and expensive surveys and studies; (4) insufficient integration of the health information system and research for health activities; and (5) duplication of statistics tasks with other Ministry of Health units. These issues were also mentioned by several participants, with one summing it up particularly well, as:

“*… the weak link between the information that is produced and the actions that are taken, or that are to be taken in the health system; the information produced by the health system is useful for promoting quality; however, the assessment of its recipients constitutes an essential source of information that is unfortunately very poorly documented, and to which special attention should be paid; the time that must be dedicated to monitoring systems seems to be excessive because there is a large number of forms to fill out and players often find the same data in several formats; the time dedicated to meetings on the health information that needs to be collected by health district officials is excessive; the absence of a directorate in charge of coordinating the Health Information System; the lack of publications; poor distribution of studies and health information; and the under-valuing of study results.*” [Senior policymaker]

#### Policies

Many policy options have been suggested to improve Burkina Faso’s National Health Information System. These included retaining the health information system in the Directorate of Studies and Planning, but increasing the staffing, equipment and resources available; the creation of a small technical directorate under the directorate of Studies and Planning; and creating a separate unit with the autonomy to manage the system. Development partners were key players in coordinating the process to prepare for the transition from a small unit to a directorate. The responses received to requests for interviews suggest that development partners think of themselves as hidden national-level players, although the perceptions among policymakers interviewed was that their role was perhaps more overt. Among the development partners, WHO, the World Bank, and the European Union were the leaders in advocating and financially supporting the Ministry of Health to progress in this transition. To inform the policymaking process, these participants commissioned several studies, most of which involved analysis of the technical feasibility of proposed changes, including creation of new units and/or strengthening the culture of statistical generation. Most proposed policy options did not require major institutional or administrative changes [36], but rather tweaks to the existing system. As large changes were not required, most proposals fit within current budgets, with the exception of the DGISS implementation. The recommendation was originally to create a small separate and independent unit, but the Minister of Health decided it would be better to create a large directorate, requiring an additional budget. The reasons for this decision were unclear, but we speculate that it could have been to emphasise the importance of the work to be done by the directorate for the health system.

#### Politics

The politics stream covers both organisational politics and more ‘party’ and international political activity. It therefore includes consideration of interest-group pressure and administrative or legislative turnover. These played key roles in placing DGISS creation on the government’s agenda. The special interest groups included development partners, the private sector, NGOs, and researchers, who voiced concerns during policy meetings about the importance of improving the health information system [37]. Ministers and Parliament also expressed interest in regulating statistical information, leading to legislation concerning the organisation and supervision of statistics activities in the public sector (Law No. 012-2007/AN of May 31, 2007) [38]. This legislation proposed a directorate or independent statistical unit within the Ministry of Health to organise and regulate statistical activities. The transition to a general directorate also required existing workers to choose between staying in the previous directorate (the Department of Planning and Evaluation in which the previous Health Statistics Unit was embedded), or move to the new directorate without knowing how that directorate would mobilise additional resources for the implementation of their activities.

The decision to create the DGISS was accelerated by a key politics-level change. In September 2008, following the appointment of a new Minister of Health who was inclined to introduce reforms, the necessary changes to create the DGISS happened quickly. This was in contrast to the previous minister, who was inclined to maintain the existing system.

### Case study 2: CT-FBR

There were several factors that led the Ministry of Health to place the creation of the CT-FBR on the decision agenda (Table 2) [15].

**Table 2:** Events linked to the creation of the unit to coordinate performance-based financing

Year	Event
2000	MDGs established
	General Assembly on Health held
	Health system reforms proposed to improve health indicators and increase the health system’s responsiveness to important health-related challenges
	Healthcare services decentralised
	National Health Policy drafted and implemented
2001	National Health Strategic Plan operationalised
	Implementation of the Health Development Support Program
2005	Midterm evaluation of the National Health Strategic Plan
2007	Midterm evaluation of the Health Development Support Program
2009	Delegation of high-level policymakers participated in a workshop organised by the World Bank in Kigali (Rwanda) on results-based funding
	Series of workshops organised and implemented to allow various health sector players to increase their knowledge of the performance-based financing policy and move towards implementing a performance-based financing system to reach Burkina Faso’s MDGs
	Recommendations from the series of training workshops were made:
	• Testing of the performance-based financing policy within the Health Development Support Program to see if it could be introduced without modifying public funding rules
	• Establishment of a department to work full-time on introducing this policy to the health system
2010	CT-FBR established in April to:
	• Help the Ministry of Health reach its goals
	• Draft the basic performance-based financing strategy/documents for the Burkina Faso health system
	• Start implementation of performance-based financing throughout the country, from January 1, 2011
	Study trip for technical unit members to Rwanda, Bamako, Ouidah, Cameroon, Senegal, and Burundi undertaken
2011	Performance-based financing test application established in three health districts to:
	• Test the capacity of the various players
	• Verify the system for the quantitative and qualitative evaluation of service providers’ performance
	• Highlight possible deficiencies in the implementation strategy in the strategy document and the national implementation guide
	• Propose possible adjustments to the implementation strategy such as those retained in the strategy document and the national implementation guide
	Actual purchasing of health interventions in three health districts (Boulsa, Léo, and Titao) began in April
2012	Test phase continued and funding sought to expand to other Ministry of Health entities
	Credit agreement signed in September between the Ministry of Finance, the Ministry of Health, and the World Bank to support Burkina Faso’s implementation of a performance-based financing project in the field of reproductive health
2013	External evaluation of the performance-based financing test phase in the Boulsa, Leo, and Titao health districts. It noted:
	• A strategy that produces good results should be adopted;
	• There was commitment and motivation from all players involved in the process
	• Ministry of Health should pursue the strategy in the three health districts and petition for its extension

*CT-FBR* Unit to coordinate performance-based financing, *MDGs* Millennium Development Goals

#### Problems

In Burkina Faso, the funding for health services is allocated by the Ministry of Finance to the Ministry of Health. The Ministry of Health then allocates funding to all the public providers in the health system, including hospitals, health districts, and central level units [39]. Allocation of funding from both development partners and the common basket is based on the activities set out in annual action plans. A small proportion (17.5%) of the total resources available to the health system is allocated based on performance against five indicators (immunisation coverage, births, caesarean sections, contraceptive prevalence, and management of referred patients) [40]. The funds linked to this performance are fully incorporated in the district action plans and providers face no personal incentives [40].

The analyses of interviews and documents concerning health finance and healthcare highlighted affordability problems for the population. Burkina Faso has no social health insurance scheme; private health insurance represents a small share of total health expenditure (less than 1%), and 97% of household health expenditure is out-of-pocket. In addition, most interview participants noted health workers’ low motivation, which contributed to a deterioration in healthcare quality. Of the 230 action plans investigated, 213 (93%) mentioned low healthcare quality as being partially a consequence of workers’ low motivation. Moreover, the use of health services was low (if increasing over time), with a health service attendance rate of 38.6% in 2006, 42.6% in 2007, and 56.6% in 2009. This highlighted the need for actors in the health system to trigger a new dynamic that served the whole system.

In February 2009, the World Bank organised a workshop in Rwanda on performance-based financing in the health sector. This workshop was organised for senior managers from Francophone African countries to encourage introduction of this policy across the region. A delegation of senior Ministry of Health figures from Burkina Faso attended. Following this trip, the attendees suggested to the Minister that performance-based financing might help to strengthen the link between performance (or quality of care) and funding in the health system, and therefore address some of these problems. Introducing such a system required a unit to support its implementation, namely the CT-FBR unit.

The creation of the CT-FBR unit was also influenced in part by the identification of problems in the 2005 evaluation of the National Health Strategic Plan [41] and various national health accounts studies [37, 42, 43]. These evaluations and studies showed an annual increase in total health expenditure, with poor performance on health indicators, poor quality of care, and a lack of health worker motivation [44]. Despite numerous initiatives implemented by the Burkina Faso government to achieve the MDGs for health, a significant gap remained between the resources used and the results achieved [45]. Various evaluations and analyses showed that the prospect of reaching the MDGs in health was poor [46].

In summary, according to several of those interviewed, the original problems leading to implementation of the performance-based financing policy and the creation of the supporting unit included poor performance on health indicators related to the use of funding, poor quality of care, and low motivation of healthcare staff.

#### Policies

Between September 22nd and October 1st 2009, a series of training workshops on the provisional performance-based financing policy was held in Ouagadougou, bringing together over 100 key policy actors in the health system. Attendees included staff from the Ministry of Health (cabinet, general secretariat, central directorates, regional health directorates, health districts, hospitals, projects), the Ministry of Economy and Finance, the Ministry of Civil Service and State Reform, the Ministry of Territorial Administration and Decentralization, development partners, and representatives of trade unions and NGOs working in the field of health. After these workshops, it was agreed that the political governance of the performance-based financing reform process should sit within the Directorate of Studies and Planning in the Ministry of Health, which would be responsible for effective coordination and monitoring of the implementation process [47].

The Ministry of Health decided to implement a national performance-based financing policy. This strategic decision was based on the favourable environment and experiences such as the Emergency Obstetric and Newborn Care subsidy, the contractual mechanism in progress, and the implementation of the 2011–2020 National Health Strategic Plan. One interviewee mentioned that the strong leadership available in the Ministry was an asset to the process. However, during initial work on introduction of the national system, in December 2010, concerns were raised about the resources available, including financial, material and human resources. It was therefore agreed that it would be helpful to carry out a testing phase in three health districts (the Titao Health District in the Northern Region, the Boulsa Health District in the North Central Region and the Leo Health District in the Central-Western Region). The test was initially to last 9 months, but in fact it started in April 2011 and did not end until December 2013. The aim of the test was to (1) enable those involved to strengthen capacity on the performance-based financing strategy and the consequent mobilisation of financial resources; (2) verify the quantitative and qualitative assessment of the providers, in particular the relevance and consistency of the selected indicators, the effectiveness, the relevance and the consistency of data collection, the performance evaluation tools, and the efficiency of the circuit of the data transmission, processing and data management; and (3) highlight possible shortcomings in the implementation of the performance-based financing strategy and propose adjustments. This pilot phase was funded with support from development partners, including the World Bank, and the extension to a further 12 health districts at the end of 2013 was funded by the World Bank.

As these difficulties became clearer, and the testing process continued, interviewees noted that the implementation of the performance-based financing policy moved higher up the agenda in the Ministry of Health [47]. Although the Directorate of Studies and Planning had been identified as having responsibility, the reforms geared toward technical governance suggested the need for a national technical unit to work full-time on this policy. The decision was therefore made to establish the CT-FBR.

#### Politics

The Ministry of Health and various interest groups, including development partners, played key roles in the politics stream for performance-based financing. Perhaps the first movers within the Ministry of Health were those who had attended the workshop in Rwanda, in February 2009; interviewees mentioned in particular the Director of Studies and Planning. This would therefore suggest that the World Bank’s influence was key in first bringing the idea to the attention of policymakers, and indeed, the funding for the project has largely come from this source. It was clear from the interviews, however, that the need to improve the health system was also important in moving the issue onto the decision agenda. Performance-based financing was seen as a way to enhance quality, counteract some of the negative effects on provider behaviour from the obligatory pre-payment schemes, and motivate the underpaid or under-motivated health workforce [48, 49].

The policy affected numerous players in Burkina Faso’s health system, including providers who received incentives and those who did not; patients who did or did not benefit from improved services; and at the national level, the program management unit that received more incentives than other programs in the same health system. Involvement of these stakeholders was critical in maximising the effectiveness of performance-based financing and minimising potential resistance that might interfere with implementation [50].

Political commitment was deemed critical to success. To obtain political support at national, regional, and district levels, stakeholders, including healthcare professionals and staff, were involved from the idea to the implementation stage. Implementation took place in several steps (e.g. meetings, training, piloting, scaling, and evaluation), and guidelines and strategic documents were developed to support the policy implementation and engage stakeholders. There was also strong commitment from government, development partners, and providers to finance the policy with budget resources and include it in the National Health Strategic Plan. Strategic documents were developed to define the pilot (conducted in three health districts), scaling budget, and stakeholder contributions to fund the process.

Parliament expressed the need to increase the accessibility of health services by delivering good services at an affordable cost. However, there was no legislative change involved in bringing performance-based funding to the decision agenda. Rather, the World Bank was the key player pushing this policy, and the idea was taken up enthusiastically by policymakers looking for a way to improve health service provision.

In summary, the creation of the CT-FBR unit was influenced by problems, policies and politics. The main problems were poor performance on health indicators, poor quality of care and poor health workforce motivation despite an annual increase in total health expenditure. Policy proposals originated from a range of participants, both visible and hidden. It is probably fair to say that the World Bank was a key player, not least because of the funding it provided. Without support from policymakers within Burkina Faso taking up the idea as a way to address problems in the system, however, the policy would not have been adopted.

### Learning from commonalities and differences

Three lessons were learned from the process of moving two key health system issues to the Ministry of Health’s decision agenda. First, the policymaking process in Burkina Faso’s health system mainly involved managers and development partners. These players influenced the agenda setting processes differently. Ministry of Health players tended to act according to their position within the Ministry and particularly whether their job role was affected by the issue. Development partners usually suggested ideas to the technical units. Within Burkina Faso, the advisers and directors of various units within the Ministry of Health were more involved in the policymaking process than any other player, and were crucial to getting acceptance for ideas, even if these ideas had first been put forward by external organisations and development partners. They participated in a number of policymaking platforms, including meetings with the minister of health, the Secretary General, and the Executive Board of the Ministerial Sector (CASEM). During these meetings, the players described problems and proposed solutions. Then, if the issue was perceived as important, it progressed to the decision agenda.

The second lesson learned was that the policymaking process in the Ministry of Health is characterised by a set of mechanisms divided into informal and formal phases. Idea formulation usually occurs during the informal phase, where hidden participants, such as development partners, start their interaction with the Ministry. An idea is typically formalised during the formal phase by the addition of comments and suggestions from visible and hidden participants. Decisions are usually made at the level of the Office of the Minister, in meetings with the Secretary General, during CASEM meetings or during council of ministers meetings.

The third lesson learned was that Ministry of Health decisions may involve institutional changes (a long process, the DGISS creation) or specific technical changes (a relatively short and simple process, the CT-FBR creation). Regardless of whether the decision is institutional or technical, policymaking follows a single channel. The idea is usually introduced by a hidden participant at the level of technical unit in charge of implementing the policy, then moves from the unit level to the Secretary General’s office. After that, technical documents articulating ideas are sent to CASEM meetings for presentation and validation. Policymaking is usually led by the Minister’s technical staff, and typically final decisions are made either by the Minister of Health or at the council of ministers meeting.

**Fig. 2:**
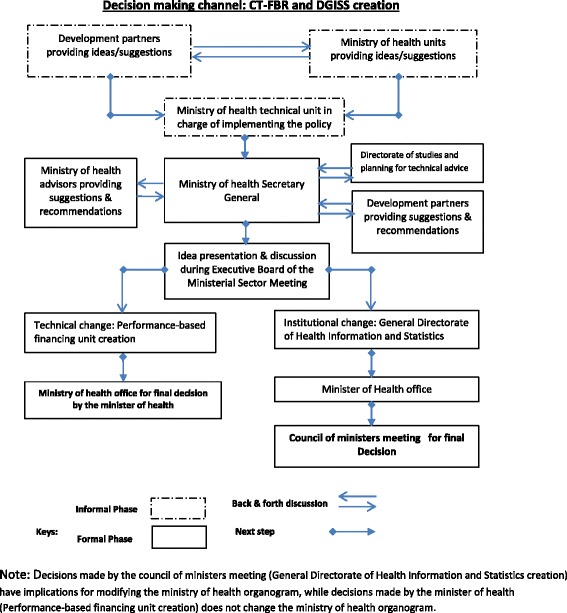
Decision-making channel: CT-FBR and DGISS creation

The final lesson learned was that the DGISS creation was mad during the council of ministers meeting and the CT-FBR by the minister of health. This process is set out in Fig. 2.

## Discussion

Our analysis of the creation of two units within the Burkina Faso Ministry of Health identified many factors that influenced the policymaking process. Kingdon’s model of agenda setting [15] was helpful in identifying the key factors that first brought issues to the attention of policymakers, and then supported decision-making. Problem, policies and politics all played a role in placing the creation of the units on the decision agenda, and then in deciding to establish them.

Policy proposals came from a range of sources, with development partners playing a key role in raising the issue in both cases. The creation of the units was significantly influenced by the opinions, interest and energy of these organisations. This is perhaps not surprising; at the international level, developing countries are often particularly exposed to global events and actions and have come to depend on development partners such as the World Bank for financial and technical assistance [51, 52]. One study noted that “*Because poor countries generally have fragile politics and weak systems of accountability, with few autonomous institutions and little power to offset that exercised by the central government, external agencies are potentially key political players, capable of exerting considerable influence*” [53]. In Nigeria, a study from 2012 stated that “*States are no more at liberty to decide their internal and domestic policies as it interests them due to the impact of international surveillance as well as actors*” [54]. A key point for both units, however, seemed to be that a critical mass of people wanted to change the system. The history of previous units in similar areas showed that one player was not enough; for example, development partner’s pressure was insufficient unless there was also some political will in the Ministry of Health. The political will tended to emerge in response to pressure from problems without a clear solution except the proposal from the development partners.

Beyond the work of development partners, one influential element in the political stream in the creation of both units was administrative changes. For the DGISS, the changes included the appointment of a new Minister of Health in 2008, as well as the MDGs and the evaluation of the 2001–2010 National Health Strategic Plan. For the CT-FBR, the change involved the establishment of a new budget allocation system based on reimbursement for services, and a pilot implemented in three districts, which made clear that a separate technical unit was required for successful policy implementation. Our findings therefore suggest that, although external ideas were important in first raising policy issues to prominence, political will and a practical need were also necessary if they are to be successfully implemented.

Kingdon’s [15] framework has previously been used to examine policy implementation in both Burkina Faso and middle-income countries, although this is the first time to our knowledge that the agenda-setting section has been used specifically to examine the establishment of a unit within the health system. In 2009, the framework was used in Burkina Faso to see whether it was helpful in examining the implementation of the Bamako Initiative health policy. The findings confirmed that the framework could be extended, leading to the formulation of several theoretical propositions [55]. The study also found that NGOs had the potential to be key players but played only a passive role because they were too dependent on development partners. This may explain why NGOs did not play a large role in our study. However, another study in Ghana using Kingdon’s framework [56] to understand and explain the agenda-setting of the Mental Health Act, found that all stakeholders played important roles in putting the issue of mental healthcare and treatment on the government agenda. In particular, NGOs played a significant role and took advantage of policy windows to push their proposals.

Also in Ghana, a study used Kingdon’s framework [15] to explain how the problem, politics and policy streams converged to enable Ghana’s National Health Insurance Scheme to become law in 2003. The study concluded that a change in government in the 2000 general election opened a ‘policy window’ for eventual policy change from ‘cash-and-carry’ to the National Health Insurance Scheme [57]. Another group used Kingdon’s [15] framework to study the development of public policies to address non-communicable diseases in the Caribbean country of Barbados. This study found that a significant policy window was opened between 2005 and 2007, enabling the Ministry of Health to create new posts to address non-communicable diseases and to establish a government-supported multi-sectoral group [58]. Similarly, in our study, the creation of the DGISS was accelerated by a key change in September 2008, when a new Minister of Health was appointed, enabling the creation of new posts.

### Strengths of the study

First, the case study method involved detailed, holistic investigation, providing a valuable way of looking at the policymaking process in Burkina Faso [52]. For this study, the case study approach was useful in describing the various factors relating to the creation of the two units. The timeline and diverse data sources also helped to describe the creation of the two units. The second strength was the case analysis and cross-case comparisons, which enabled a number of overall themes, concepts and relationships to be characterised during analysis. The ability to identify broader themes, concepts and relationships through multiple and cross-case comparisons ensured a robust understanding of the agenda-setting stage in the policymaking process in Burkina Faso’s health sector. This study builds on previous studies using Kingdon’s framework, and confirms its usefulness as a methodology and conceptual framework for this type of analysis.

### Study limitations

This study had several limitations. First, interviewing development partners was not possible for various reasons. For example, the development partners referred us to the Ministry of Health, arguing that they were not direct players in policymaking but only facilitators of the process through provision of financial support for implementation. Interviewing development partners would have enabled us to explore the reasons underlying their recommendations in particular policies, which were not clear to national-level policymakers, and therefore deepened the study considerably.

Second, some of the policymakers interviewed were still working at the Ministry of Health. They knew the first researcher well, and so were comfortable discussing events that occurred during the policymaking process, and expressing their opinions about why decisions had been made. However, they often asked for their opinions not to be transcribed or published, even if others also expressed the same view, in case it caused problems with senior policymakers still working in the healthcare system. It is therefore possible that some widely-held views about the reasons for particular decisions have been excluded from this study. To overcome this problem, as well as using the same questions for several interviewees, we also drew on the personal experience of several of the researchers, including one who has been working in Burkina Faso for more than 25 years, enabling us to triangulate the data more successfully.

Thirdly, in Burkina Faso, given that policymaking generally involves high-level players, many of the key informants identified were busy and it was often a challenge to make interview appointments, particularly for the more senior policymakers, even drawing on the principal investigator’s personal relationships and knowledge. More detailed interviews with more senior policymakers might have exposed an increased level of detail about the thinking behind the policy, and enriched the analysis, particularly in the absence of any interviewees from development partners.

It is also possible that participants’ recall was affected by the time between the events being discussed, many of which took place in 2009 or earlier, and the interviews themselves in 2013. Participants may therefore have been providing views that they only developed later, or may have forgotten what had actually happened.

We are aware that there may have been some connections between the decision-making processes around the two units. However, we have treated them as separate in this study because neither interviewees nor documentary evidence suggested that these links existed. However, it is possible that these links were so ingrained that nobody mentioned them, and if so, this might affect the establishment of similar units elsewhere.

Finally, the involvement of the principal investigator was both a strength and a weakness of the study. Having been involved in some of the discussions around establishing both units, he had a knowledge and understanding of the process, and also knew most of those involved. This meant that participants were prepared to talk openly, but also that some implicit knowledge may not have been surfaced or explored, and there may have been some hidden bias [58]. As far as possible, this was eliminated by involving other researchers in the process, especially another researcher who was very familiar with the setting and location.

### Implications for policy and practice

To successfully institutionalise a unit, key factors to consider include a well-understood problem, a viable policy, and conducive politics. While the specifics in each of these streams differed across the two cases, Kingdon’s framework provides a useful guide in what to look for when trying to move an issue to the decision agenda.

### Implications for future research

Future research should continue to use Kingdon’s framework [15], but extend its application to more cases and include interviews with a broader array of civil servants, development partners, and health system workers. In particular, researchers should seek to interview high-level policymakers and representatives from development partners to improve understanding of the thinking behind policy proposals. This information is often unknown to more junior staff. Future research could also broaden the focus beyond agenda-setting to study the factors that shape policy choice itself (i.e. the existing institutional, interest-related, and ideological factors that shape policy development).

## Conclusions

Three streams – problem, policy, and politics – influenced the move of the two units onto Burkina Faso’s decision agenda, resulting in decisions to create the units. The problem stream was marked by a critical mass of people interested in changing Burkina Faso’s health information and performance systems, for a variety of reasons. These included both external (often development partners) organisations, and also policymakers from within the Ministry concerned about how to address major policy problems. Both these groups put forward policy proposals, and it seems likely that it was at least partly the coincidental aligning of interests that made implementation possible. In both cases, the political stream was marked by changes in political and public service administrations. For the DGISS, this started with the appointment of a new Minister of Health, and for CT-FBR, it coincided with the establishment of new budget allocation system. This suggests that the timing of policy implementation is crucial in achieving success. Those charged with establishing similar units in future may need to consider issues of timing, as well as gathering a critical mass of supporters. They may also need to look beyond agenda-setting, to policy implementation and issues of institutionalisation such as long-term funding, a government mandate to implement the policy, and how the unit will help to resolve problems raised by the health system.

## Appendix 1

**Table 3:** Details of study participants’ job roles and level of experience

	ID	Job roles	Years of experience
National Health Information System (DGISS unit)	1	Senior policymaker	22
	2	Senior policymaker	8
	3	Senior policymaker	8
	4	Senior health district manager	7
	5	Senior health district manager	16
	6	Programme manager	17
	7	Programme manager	19
	8	Senior policymaker (regional)	21
	9	Finance manager	12
	10	Senior policymaker/programme director	16
	11	Senior policymaker (regional)	12
	12	Senior policymaker	25
	13	Programme manager	11
	14	Project coordinator	16
CT-FBR	1	Unit staff	17
	2	Unit staff	13
	3	Senior policymaker	22
	4	Finance manager	11
	5	Finance coordinator	19
	6	Senior policymaker	23
	7	Policymaker	13
	8	Policymaker	13
Ministry of Health general administration	1	General hospital director	16
	2	Senior policymaker	26
	3	Policymaker	11
	4	Health economist	12
	5	Senior policymaker	15
	6	Senior policymaker	18

Note: To preserve confidentiality, participants have been categorised by level of seniority and job roles. Where helpful, regional input or particular expertise has been shown

## Appendix 2. Interview guide used during the study

Analyse the process of policymaking in the health sector of Burkina Faso and in turn document the factors that influence the use of scientific evidence in the health policymaking process.

### Section 1 – General information

1. Could you introduce yourself? How many years of experience do you have in the health system and in policymaking?
2. How do you perceive your role in the policymaking process and can you describe the policymaking process in the Ministry of Health in general?

### Section 2 – Knowledge in policymaking

3. Please will you tell me about your experience of the creation of the performance-based financing unit in the Ministry of Health (CT-FBR) and/or the Division of Heath Information and Health Statistics (DGISS)?
4. What was your involvement and what role did you play?
5. Did you call upon external actors? If yes, why and how? Explain the role played by those external actors in the policymaking process.
6. What strategies were used to get the issue onto the government agenda?
7. Do you think the media played any role?
8. Do you think elections or political change played an influential role in the creation of DGISS or CT-FBR?
9. Did a change in government influence the agenda-setting process?
10. How significant was the role of civil society organisations?
11. Did you face challenges? If yes, what were some of them?
12. How were you able to address these challenges?
13. How would you assess the weight of the views of particular actors during the process? (Examples).
14. What happened before the unit was established? Did you organise trips to other countries beforehand, for example?
15. During the policymaking process, for each of the units, do you think its implementation was influenced by external actors (development partners, NGOs, society, or others); can you describe the contribution of each of the stakeholders?
16. Do you think that the Ministry of Health chose to implement these policies to be like other countries?
17. Can you explain how useful the units are in terms of their additional value for the system.

### Section 3 – Identifying the Problem

18. Did you carry out scientific studies before describing the problem? If yes, can you explain the process? If no, why not?
19. Have you got any idea of the issue that motivated the introduction/suggestion of the unit’s establishment?
20. Do you think that you got enough information (objective or subjective) to describe the issue?
21. Do you think that you could have improved the description of the issue? At what level did you need policy information?
22. Did you have enough time and resources to define the issue?
23. Were you able to assess the advantages and disadvantages of each solution proposed?
24. Were you able to establish criteria to assess the solutions?
25. What criteria guide you in policymaking? Grade these from most important to least.
26. During policymaking, did you use scientific evidence from research results to shed light on the policies? If yes, describe how you got it.

### Section 4 – Implementation of the policy

27. Were you able to define the actions required to achieve the objectives?
28. Do you think that the units were established as described in the texts/legislative policies? If yes, explain how.
29. What do you know about public opinion on the policies?
30. Do you think that the unit contributes to achieving the objectives expected? If yes, how?
31. Do you think that the unit was implemented adequately?
32. Do you think that the unit really benefits the population?
33. Describe the budget allocation and source of funding for DGISS and CT-FBR implementation.

## Abbreviations

ARCS: National Program for Strengthening Statistics System Capacity
CASEM: Executive Board of the Ministerial Sector
CT-FBR: Unit to coordinate performance-based financing
DGISS: General Directorate of Health Information and Statistics
MDGs: Millennium Development Goals
NGOs: Non-government organisations
